# Social determinants of health in non-communicable diseases prevention policies in South Africa

**DOI:** 10.4102/curationis.v46i1.2387

**Published:** 2023-08-22

**Authors:** Richard M. Rasesemola, Rose M. Mmusi-Phetoe, Yolanda Havenga

**Affiliations:** 1 Department of Nursing, Faculty of Health Sciences, University of Johannesburg, Johannesburg, South Africa; 2 College of Human Sciences, University of South Africa, Pretoria, South Africa; 3 Faculty of Science, Tshwane University of Technology, Pretoria, South Africa

**Keywords:** social determinants of health, non-communicable diseases, policy analysis, qualitative document analysis, intervention strategies

## Abstract

**Background:**

The South African government has developed many policies for the prevention and control of non-communicable diseases. However, non-communicable diseases remain among the major causes of morbidity and mortality in South Africa. Although these diseases are linked to interaction of multiple risk factors, many of which are modifiable, they continue to cause much suffering particularly among the marginalised and people from the lower socio-economic status.

**Objectives:**

The objective of this research was to explore and present the inclusion of social determinants of health in the policies meant for the prevention and control of non-communicable diseases in South Africa.

**Method:**

The qualitative document analysis approach was used to conduct policy analysis of purposefully selected policies for prevention and control of cancers, obesity and mental and behavioural disorders in South Africa.

**Results:**

The analysis revealed that policies for prevention and control of cancers, obesity and mental and behavioural disorders included policy intervention activities that focused on five social determinants of health: (1) governance, (2) social policies, (3) public policies, (4) material circumstances and (5) health system.

**Conclusion:**

Excluding most of the important social determinants of health in the policies for prevention and control of non-communicable diseases means that these policies would continue to fail in preventing these diseases from the root causes.

**Contribution:**

This article points out weaknesses in the policies meant for prevention and control of obesity, cancers and mental and behavioural disorders. This article further suggests policy improvement strategies that may be considered to effectively address these diseases.

## Background

Over 70% of 56 million global deaths were attributable to non-communicable diseases (NCDs), with over three quarters occurring in low- and middle-income countries (World Health Organization [WHO] 2018). Despite the known risk factors and the preventable burden on healthcare systems posed by NCDs (WHO 2021), NCDs are the biggest cause of mortality globally compared to the other diseases (Spires et al. 2016). The number of deaths from NCDs is expected to increase drastically over the coming years around the world (Wang & Wang 2020). The greatest increase is expected to be in the sub-Saharan African region (Gouda et al. 2019).

To begin with, the disproportionately larger burden of disease because of NCDs is more prevalent in sub-Saharan Africa and other low-income regions because of poverty (WHO 2020). In addition, the likelihood of dying from NCDs in these regions is twice as high compared to that of higher-income regions (WHO 2018). Furthermore, the trend is similar in the Southern African Developmental Community region where most of the risk factors for NCDs and poor health outcomes from NCDs tend to cluster more among the poor communities than in their affluent counterparts (Umuhoza & Ataguba 2018). Likewise, in South Africa, NCDs and related risk factors also tend to cluster among the poor communities (Wekesah et al. 2018), with those living in the rural areas being mostly affected (Juma et al. 2019).

Social determinants of health determine these differences between the low- and high-income countries in the prevalence of NCDs and the likelihood of dying prematurely because of NCDs (WHO 2019). Social determinants of health refer to the systems that influence the conditions of daily life, as well as the living and working conditions that influence health risks and functioning (Castrucci & Auerbach 2019; WHO 2019). Social determinants of health influence health outcomes in several ways. To begin with, social determinants influence the distribution of the main behavioural risk factors of NCDs including unhealthy diet, physical inactivity, tobacco smoking and excess alcohol consumption (Arquilla & Sherrell 2021). In addition, social determinants of health influence the physical conditions that are risks for the development of NCDs including raised blood pressure, obesity and diabetes (Marmot & Bell 2019). Furthermore, social determinants influence social conditions including poverty experienced from before birth and in early life which have long-lasting effects during a lifetime and contribute to the development of NCDs (Marmot & Bell 2019).

Poverty experienced in the early years affects the development of parts of the brain that contribute to the cognitive control over diet and physical activity levels (Marmot & Bell 2019). Poor development of these areas of the brain that regulate behaviour and thought is further linked with people’s capacity to resist smoking, excessive alcohol consumption and other unhealthy behaviours (Marteau & Hall 2013). This association contributes to higher rates of smoking, drinking, poor diet and physical inactivity that give rise to the differences in life expectancy between the least and most deprived sections of society. Subsequently, people from lower socio-economic class have lower cognitive control over their diet and physical activity levels leaving them at increased risk of developing NCDs (Marmot & Bell 2019). In addition to the poor cognitive control over food and physical activity, because of poverty and inability to afford healthy meals, people opt to consume energy-dense food that is cheap and gastronomically filling instead of consuming a healthy diet (French et al. 2019). As a result, inequalities in terms of vulnerabilities to and health outcomes from various NCDs ensue as people living in poverty are restricted in their ability to practise healthy behaviour (Niessen et al. 2018).

These inequalities further include the unequal distribution of income, resources, health care services and circumstances of people’s lives (Marmot & Bell 2019). The inequalities in people’s material living standards include the physical environment, type of housing and its location, access to healthy food and working conditions linked with mental and behavioural disorders (Solar & Irwin 2010). These disorders further affect health through unhealthy behaviour such as eating fatty foods, comfort eating with a diet rich in carbohydrates, smoking, excessive alcohol consumption and a lack of physical exercise (Lightfoot et al. 2018).

Besides people’s socio-economic status and social stratification, political context determines people’s access to resources and structural and functional aspects of a social system that influence health opportunities and vulnerabilities to NCDs (De Andrade et al. 2015). The policy decisions influenced by the political context to structure the society according to race, gender and age-based hierarchies during the South African apartheid government left enduring effects of differences in access to healthcare services and health outcomes between black and white people, and the rich and poor (Coovadia et al. 2009). The effect can be seen in the differences in terms of life expectancies between South African white women and black women, with the latter being the worst off (Mhlanga & Garidzirai 2020). In addition, the effect can be seen in the differences in terms of the mortalities from NCDs between black people and white people (Mhlanga & Garidzirai 2020).

Addressing these inequalities, vulnerabilities to NCDs and related health outcomes, it is necessary to address the origins of factors that introduce, facilitate and reinforce the emergence and sedimentation of these inequalities (Van Rensburg & Fourie 1994). So, addressing these inequalities, and prevention and control of NCDs requires comprehensive policies that would ensure that efforts are directed towards addressing the social determinants of health.

To address the inequalities in the development of NCDs among the South Africans, several strategies and policies were developed. Such strategies were meant to ensure the desirable population-wide health outcomes and reduce vulnerabilities to the NCDs. Yet, more than 50% of deaths in South Africa in 2020 resulted from NCDs (WHO 2020). As a result, not much was achieved from such strategies and policies in terms of reducing the burden of disease because of NCDs.

### Research aim

Among the NCDs that contributed to most deaths are the mental and behavioural disorders; cancers and conditions that have some links with obesity such as endocrine, nutritional and metabolic diseases; circulatory system disorders (WHO 2020). While some NCDs have been declining, these conditions have been on the rise in South Africa between, notably from 2015 (Department of Statistics South Africa 2017). Therefore, this article intends to present the inclusion of social determinants of health in the policies meant for the prevention and control of NCDs in South Africa, specifically obesity, behavioural and mental health disorders and cancers. The specific aim of the study was to present and provide strategies that could be used to accelerate the country’s response to the prevention and control of NCDs.

## Research design and methods

Qualitative document analysis was used for policy analysis in this study. Qualitative document analysis is a form of research in which documents are interpreted to give them voice and meaning around the phenomenon of interest (Bowen 2009:29). Qualitative document analysis as a research design is conducted in a way that is similar to how researchers would approach qualitative transcripts and it involves coding content from the documents into themes (Bowen 2009:29). In this study, the researcher used qualitative document analysis by developing the criteria which was systematically applied to the NCD policy documents. Thus, using qualitative document analysis, the researcher ‘asked’ the questions from the policy documents that need to be asked and ‘observed’ situations and conditions that need to be observed.

### Population and sampling

Population included policies for the prevention and control of NCDs in South Africa. Purposive sampling technique was used to identify three policy documents for inclusion in the analysis. Such policies included the health policies meant for prevention and control of cancers, obesity, and mental and behavioural disorders in South Africa. These policies were purposefully selected and included in the policy analysis because of the increasing burden of disease in South Africa which they are meant to prevent and control. The policies are publicly available and were obtained through electronic searches on the Department of Health’s website.

### Data collection

Data collection was conducted in steps using a common set of criteria that was developed by the researcher and systematically applied to the policy documents being analysed, focusing on the following aspects:

**Step 1:** Identification and description of the policy document.

**Step 2:** Identify the policy background and agenda identify stakeholders; explore and describe the social determinants of health in the implementation plans and activities.

**Step 3:** Evaluate the policies by comparing identified intervention activities and their targets with evidence from literature.

### Data analysis

Data analysis was conducted using inductive content analysis guided by the study’s objectives. Inductive content analysis is a systematic procedure of analysing qualitative data, where the analysis follows the process of abstraction to reduce and group data so that researchers can answer the study questions using themes guided by specific objectives (Kyngäs 2020:13). Inductive content analysis requires the researcher to find links between the research objectives and the findings derived from the documents chosen for analysis (Kyngäs 2020:13; Thomas 2006:238). In this study, the researcher identified the social determinants of health and health inequalities from the analytical framework for social determinants of health (Solar & Irwin 2010) and the implementation activities from the policy documents were then classified according to the social determinants of health and health inequalities. Therefore, the findings arose directly from the analysis of the raw data (policy documents) but were influenced by the objectives of the analysis (Thomas 2006:238).

### Trustworthiness

To uphold trustworthiness of qualitative document analysis in this study, the four principles including authenticity, portability, precision and impartiality were followed (Morgan 2022). These are disciplinary standards that apply in qualitative document analysis and should be adhered to when treating texts as data to establish legitimacy of the research by protecting its trustworthiness (Morgan 2022).

### Authenticity

Authenticity is the truth value of the research. An authentic qualitative document analysis offers an accurate reading and genuine interpretation of the policy documents. Authenticity of qualitative document analysis relies upon the subjective evaluation of the researcher, instead of being based on some objective standard. To ensure authenticity and that the researcher’s interpretation is a true reflection of the contents of the policy documents, consensus meetings were held with an independent coder to establish consensus relating to the findings.

### Portability

The researcher provides a comprehensive description of the documents analysed, data-collection procedure, and data analysis and interpretation procedures followed, to enhance the possibility of other researchers repeating the study. To ensure portability the researcher provided comprehensive information related to the procedures followed in policy analysis as well as making linkages between the findings of the current studies with evidence-based literature.

### Precision

Researchers involved in qualitative document analysis should ensure precision in their analysis. This is known as dependability, and it refers to the stability of data over time and conditions. Dependability ensures that the findings could be replicated should the study be repeated with the same documents under similar conditions (Roux 2002). It is when the policy analysis has satisfied the requirements for audit trail, code-recode procedure and audit strategies that it is deemed dependable. Therefore, to ensure precision in this research, the researcher followed well-established procedures and processes that have previously been used in policy analysis studies.

### Impartiality

Researchers involved in qualitative document analysis are believed to produce unbiased knowledge about the social world through findings that are reflective of reality instead of their own pre-determined beliefs. This means preserving the objectivity of the analysis (Taylor, Haglund & Tillgren 2000). To ensure impartiality, the researcher had to achieve confirmability of findings, ensuring that conclusions are drawn from the evidence from the policies as opposed to preconceived predispositions. Conformability of a study is considered to have been achieved when credibility, audit trail and transferability can be demonstrated. In this study, conformability was adhered to by ensuring that the results are confirmable by drawing inferences and substantiating with evidence from the policy documents so that the process can be traced.

## Results

Policy analysis in this study included three policy documents; National Cancer Strategic Framework for South Africa 2017–2022, Strategy for the prevention and control of obesity in South Africa 2015–2020, as well as National Mental Health Policy Framework and strategic plan 2013–2020. Following the identification and description of the current health policies interventions for the prevention and control of NCDs in South Africa, the researcher described the policy agenda and stakeholders involved in the policy formulation. The process yielded multiple stakeholders included in the formulation of each of the three policy documents and the findings are reflected in Table 1. The researcher located social determinants of health in the key implementation activities.

**TABLE 1:** Documents included in the policy analysis.

Title of the document	Stakeholders involved in policy formulation
Strategy for the prevention and control of obesity in South Africa 2015–2020	Department of Health: ■ Health Promotion Directorate ■ NCDs Directorate ■ Nutrition Directorate Department of Transport Department of Arts, Sports, and Recreation Public Service and Administration
National Mental Health Policy Framework and Strategic Plan 2013–2020	Department of Health Department of Education Department of Social Development Department of Correctional Services Department of Justice Department of Transport Local government South African Police Services Civil society
National Cancer Strategic Framework for South Africa 2017–2022	Department of Health ■ Ministerial Advisory Committee of Cancer Civil society Academic and clinical experts National Cancer Registry Global health organisations

NCD, non-communicable diseases.

Table 1 includes three documents that were identified and analysed in this study. As the custodian of health in the Republic of South Africa, the policy documents had to be produced or endorsed by the Department of Health in South Africa to be included in the study. These documents were obtained electronically from the Department of Health’s website.

### Strategy for the prevention and control of obesity in South Africa 2015–2020

The strategy document was developed with the purpose of identifying gaps and reviewing current policies and regulations, as well as implementing a multisectoral approach for the prevention and control of obesity in South Africa. This strategy document recommends a population-based approach that is centred on policy and environmental changes. The objective of this strategy is to reform obesogenic environments and enablers, while enhancing opportunities for increased physical activity and healthy food options in various settings including healthcare facilities, early childhood development centres, schools, workplaces and the community.

The strategy for the prevention and control of obesity in South Africa document was developed by various health directorates and ministries and was also endorsed by other ministries such as Education, Cooperative Governance and Traditional Affairs, and Trade and Industry.

### Stakeholders involved in strategy formulation

The Strategy for the prevention and control of obesity in South Africa 2015–2020 was formulated through collaborative work of the various directorates from the National Department of Health such as Health Promotion, NCDs and Nutrition. Other stakeholders include the Department of Transport, Department of Arts, Sports, and Recreation, Public Service and Administration and the Department of Public Service and Administration; Departments of Education, Cooperative Governance and Traditional Affairs and Trade and Industry also pledged their support by endorsing the strategy document.

### Social determinants of health in the policy implementation activities

Themes identified from the strategy for the prevention and control of obesity in South Africa 2015–2020 key implementation activities are presented. The themes, which are social determinants of health and health inequalities with their supporting excerpts are presented in Table 2.

**TABLE 2:** Social determinants of health located in the strategy for the prevention and control of obesity in South Africa 2015–2020.

Determinant	Excerpt
Governance	Identify stakeholders and sectors. Explore and/or formulate a coordinating structure to guide the implementation of the strategy and agree on roles and responsibilities.
Social policies	Influence fiscal policies related to sugar-sweetened beverages. Develop norms and standards on sugar and fat content in ultra-processed foods to guide product reformulation.
Public policies	Incorporate explicit obesity prevention and control messages in ECD policies and guidelines. Review and implement nutritional guidelines for all food and beverages sold or provided in schools, including foods sold by vendors around school premises. Support and reinforce implementation of existing policies and initiatives to promote, protect and support breastfeeding.
Material circumstances	Expand household, local and community food gardens. Increase equitable access to and maintenance of recreational and physical activity facilities in communities. Increase equitable access to public transportation.
Health system	Develop a step-by-step guide on prevention and control of obesity in children for healthcare personnel. Strengthen obesity referral systems for nutritional assessment and counselling. Promote BMI assessment and counselling on obesity in healthcare settings.

ECD, early childhood development; BMI, body mass index.

The strategy for the prevention and control of obesity in South Africa 2015–2020 included social determinants such as governance, social policies, public policies, material circumstances as well as health system in its key implementation activities.

### National Mental Health Policy Framework and Strategic Plan 2013–2020

The Mental Health Policy and Strategic Plan 2013–2020 is the culmination of several processes and activities that were undertaken over time as well as review of existing mental health policies, services and systems. It was developed following a meeting with stakeholders in mental health at the 2012 Mental Health Summit and review the state of mental health services in all nine provinces. The summit resulted in the adaptation of South African Ekurhuleni Declaration on mental health, which paved a way for the Mental Health Policy Framework and Strategic Plan 2014–2020. It is the first national mental health policy to be officially endorsed. The policy aligns with and builds upon the WHO’s Mental Policy, Plans and Programmes and the Department of Health 10-point plan, and the *Mental Healthcare Act No. 17 of 2002*. The purpose of the Mental Health Policy and Strategic Plan is to give guidance to provinces for mental health promotion, prevention of mental illness, treatment and rehabilitation. The objectives of this policy include to make mental health services widely available and accessible, reduce stigmatisation and discrimination associated with mental illness, promote multisectoral approach to promote mental health through community engagement and collaboration between the Department of Health and other relevant sectors and adopt a multisectoral approach to tackle the perpetual cycle of poverty and mental ill-health in South Africa.

### Stakeholders involved in the formulation of the policy

The National Mental Health Policy Framework and Strategic Plan 2013–2020 resulted from a review of the current mental health policy and literature, as well as the situation analysis of the current mental health system in South Africa. The strategy was formulated by the Department of Health in consultation with civil society and various stakeholder from the government including the Department of Education, Department of Social Development, Department of Correctional Services, Department of Justice, Department of Transport, Local Government as well as South African Police Services.

### Social determinants of health in the document

The Mental Health Policy and Strategic Plan 2013–2020 key implementation activities included the social determinants of health as reflected in Table 3.

**TABLE 3:** Social determinants of health located in the Mental Health Policy and Strategic Plan 2013–2020.

Determinant	Excerpt
Health system	Build/attach mental health inpatient units to designated district and regional hospitals. Revitalise dilapidated mental health facilities in all provinces. Equip designated clinics and community health centres with psychology infrastructure. Make all psychotropic medicines, as provided on the essential drugs list (EDL) available at all levels of care, including PHC clinics.
Governance	Establish Mental Health Directorates in each of the nine provinces. Mental health will be included on the agenda and mental health representation will be assured on the newly established National Health Commission.

PHC, primary healthcare.

Governance and the health system appeared to be the only social determinants of health included in the Mental Health Policy and Strategic Plan 2013–2020 key intervention activities as reflected in Table 3.

### The National Cancer Strategic Framework for South Africa 2017–2022

The National Cancer Strategic Framework was developed by the Department of Health to provide guidelines for cancer prevention and treatment in South Africa. The strategic framework was developed to assist the government to ensure equitable access to cancer-care throughout the country. It is intended to guide the development, implementation, monitoring and coordination of cancer policies and strategies and it is for use by a variety of stakeholders such as the national and provincial departments of health, private sector, academia and non-profit organisations (NPOs).

The objective of the National Cancer Strategic Framework is to reduce the burden placed on the South African population as a result of cancer-related deaths, disability and financial strains. Furthermore, the strategic framework is intended to assist in the development of comprehensive cancer policies and services to address inequity in access to early detection, timely and appropriate treatment, including pain relief and palliative care in South Africa.

### Stakeholders involved in strategy formulation

The National Cancer Strategic Framework is a product of the Department of Health’s Ministerial Advisory Committee of Cancer with consultation with civil society, academic and clinical experts, National Cancer Registry, as well as global health organisations.

### Locating social determinants of health in the document

The National Cancer Strategic Framework for South Africa 2017–2022 key implementation activities included the social determinants of health as reflected in Table 4.

**TABLE 4:** Social determinants of health located in the National Cancer Strategic Framework for South Africa 2017–2022.

Determinant	Excerpt
Health system	Define referral pathways. Refine plan for multi-disciplinary oncology human resources to strengthen services, training, and specialisation.
Public policies	Development of clinical guidelines for breast cancer. Publish policies for remaining four priority cancers. Launch of the palliative, breast, cervix, and national cancer strategy framework. Develop and implement provincial plans to improve adult and children and adolescents’ oncology services to detect diagnose and treat cancer. Implementation of approved cancer and related policies.

The National Cancer Strategic Framework included health system and public policies as social determinants of health in the key intervention activities as reflected in Table 4.

Policy analysis of the three policy documents for the prevention and control of NCDs in South Africa indicated the inclusion of governance, social policies, public policies, material circumstances, as well as the health system as the only social determinants of health in their implementation activities.

## Discussion

Non-communicable diseases are driven by complex interaction of numerous and multifaceted risk factors (Budreviciute et al. 2020). These factors are known as the social determinants of health and include the structural determinants of social determinants of health inequalities and the intermediary determinants of social determinants of health (Artiga & Hinton 2018). The structural determinants of social determinants of health inequalities include socio-economic and political context including governance, cultural and social values, macroeconomic policies, social policies, public policies and the people’s socio-economic position in society such as social class, gender, race, education, occupation and income (Marmot & Bell 2019). The intermediary determinants of social determinants of health include people’s material circumstances such as living conditions and food availability, behavioural and biological factors and the health system (Artiga & Hinton 2018).

The policy implementation activities of the policies meant for the prevention and control of obesity, cancers, mental and behavioural disorders in South Africa addressed some social determinants of health including governance, social policies, public policies, material circumstances and the health system. The social determinants such as culture and societal values; and socio-economic position including gender, ethnicity, education, income and occupation, as well as the material circumstances such as living and working environment; behavioural and biological factors and psychosocial factors were excluded from these policy implementation activities. The exclusion of these social determinants of health, which are known to have strong links with the development of NCDs means that the policies for prevention and control of obesity, cancers and mental and behavioural disorders do not sufficiently address the risk factors of these diseases from their origins. Implications of such exclusion is the perpetual inequalities in health outcomes seen between people of different socio-economic statuses, race and educational achievement among others (Gordon, Booysen & Mbonigaba 2020), and the continuing burden of disease because of obesity, cancers and mental and behavioural disorders in South Africa.

Therefore, to effectively address the NCDs, the government should seek to address the differences in socio-economic, material circumstances, gender, race and educational differences from the roots by addressing circumstances and policies that enable these risk factors. To begin with, people from lower socio-economic status experience restrictions in their ability to practice healthy behaviours that promote healthy lifestyle. An example would be the link between socio-economic status and NCDs as manifested directly and indirectly through the effects on the access to healthcare facilities and preventative medical examinations (Çiğdem 2019). The restrictions in access and utilisation of prevention and care services for the poor communities relate to inability to afford transport to healthcare facilities (Burger & Christian 2020). With the crumbling state of public infrastructures that caters for more than 84% of the South African population, shortage of human resources, and a lack of essential drugs complicate the challenges and contribute to barriers in accessing healthcare services for poor South Africans (Mutwali & Ross 2019).

In addition, poverty results in restrictions in accessing proper shelter, resources used in the prevention of risk factors for NCDs and restrictions in opportunities to adopt healthy lifestyle behaviours such as appropriate nutrition (Czapp & Kovach 2017). The resulting consequences include poor cognitive functioning, lower educational achievement, increased risks of low income, unstable job status, smoking, alcohol abuse, physical inactivity and an unhealthy diet (Oshio & Kan 2020), factors associated with NCDs. These factors further exert a great influence on health because they determine material circumstances, and the type of housing and its location (Corburn & Sverdlik 2017). In South Africa, the influence of these factors is manifested with substandard housing infrastructure with poor ventilation, low availability of fresh produce because of lack of land, concentration of fast-food outlets and fewer recreational opportunities.

In conclusion, the country’s policies, including the labour market, the educational system, political institutions and other cultural and societal values, as well as social and political mechanisms generate and shape the social hierarchies and determines health outcomes from NCDs (Solar & Irwin 2010). These factors further influence a variety of social determinants of health including socio-economic factors, material circumstances, psychosocial circumstances, behavioural and biological factors and the health system to shape the population’s health outcomes (Bravemann & Gottlieb 2014). The countries with public and social policy goals that include intentions for the improvement of population health with policies that seek to promote employment and equity, increase access to equal education, provide appropriate housing, and improve health care services among others are more likely to yield positive gains towards addressing the social determinants of health and improve population health and wellbeing (Kruk et al. 2018).

### Strengths and limitations

Purposive sampling enabled the researcher to select relevant policy documents rich with information with a particular focus on prevention and control of cancers, obesity, mental and behavioural disorders in South Africa. However, the limitation of this study refers to its inclusion of policy documents for prevention and control of these specific diseases in South Africa. Thus, the findings do not refer to any other NCDs in the country and the government’s performance towards achieving targets related to addressing such NCDs.

### Implications and recommendations

The analysis revealed that policies for the prevention and control of cancers, obesity and mental and behavioural disorders in South Africa were crafted and articulated to match the WHO’s recommendations on management of NCDs; however, some important social determinants of health contributing towards the development of these diseases were not addressed. The implications include the availability of well-crafted policies that would continue to not make a measurable impact in terms of addressing NCDs and the obvious differences between the policy goals and the current mortality rates because of NCDs in South Africa. Therefore, the study recommends that it is important for policymakers to recognise that risks and development of NCDs across the population are driven by a variety of social determinants of health. Thus, to make a difference in terms of addressing NCDs in South Africa, policymakers and various relevant stakeholders should focus on developing customised-to-context type of policies that clearly articulate intentions to address all the social determinants of health, including various forms of inequalities between people of different race and gender, socio-economic status, educational differences, material circumstances such as housing and food security and biological and behavioural factors. Failure to do so would mean that NCDs would continue to lead in the causes of natural deaths in South Africa and will continue to do so unless all the social determinants of health are addressed.

## Conclusion

The analysis of policies for prevention and control of NCDs in South Africa indicated that some of the social determinants of health, as the root causes of NCDs, are not addressed. Therefore, the strategic direction for policies meant to address the prevention and control of NCDs should work towards tackling these social determinants of health with a particular emphasis on addressing various forms of inequalities and exposures. Thus, the policies for the prevention and control of NCDs should not be limited to addressing a few social determinants of health but include all the social determinants of health that systematically produce an inequitable distribution of risk factors among population groups.
